# Marital status is an independent prognostic factor for tracheal cancer patients: an analysis of the SEER database

**DOI:** 10.18632/oncotarget.12809

**Published:** 2016-10-21

**Authors:** Mu Li, Chen-Yang Dai, Yu-Ning Wang, Tao Chen, Long Wang, Ping Yang, Dong Xie, Rui Mao, Chang Chen

**Affiliations:** ^1^ Department of Thoracic Surgery, Shanghai Pulmonary Hospital, Tongji University School of Medicine, Shanghai, People's Republic of China; ^2^ Deloitte and Touche Financial Advisory Services Limited, Shanghai, People's Republic of China; ^3^ Division of Epidemiology, Department of Health Sciences Research, Mayo Clinic, Rochester, MN, USA

**Keywords:** tracheal cancer, marital status, socio-economics, SEER, survival analysis

## Abstract

**Background:**

Although marital status is an independent prognostic factor in many cancers, its prognostic impact on tracheal cancer has not yet been determined. The goal of this study was to examine the relationship between marital status and survival in patients with tracheal cancer.

**Results:**

Compared with unmarried patients (42.67%), married patients (57.33%) had better 5-year OS (25.64% vs. 35.89%, *p* = 0.009) and 5-year TCSS (44.58% vs. 58.75%, *p* = 0.004). Results of multivariate analysis indicated that marital status is an independent prognostic factor, with married patients showing better OS (hazard ratio [HR] = 0.78, 95% confidence interval [CI] 0.64–0.95, *p* = 0.015) and TCSS (HR = 0.70, 95% CI 0.54–0.91, *p* = 0.008). In addition, subgroup analysis suggested that marital status plays a more important role in the TCSS of patients with non-low-grade malignant tumors (HR = 0.71, 95% CI 0.53–0.93, *p* = 0.015).

**Methods:**

We extracted 600 cases from the Surveillance, Epidemiology, and End Results (SEER) database. Variables were compared by Pearson chi-squared test, *t*-test, log-rank test, and multivariate Cox regression analysis. Overall survival (OS) and tracheal cancer-specific survival (TCSS) were compared between subgroups with different pathologic features and tumor stages.

**Conclusions:**

Marital status is an independent prognostic factor for survival in patients with tracheal cancer. For that reason, additional social support may be needed for unmarried patients, especially those with non-low-grade malignant tumors.

## INTRODUCTION

Tracheal cancer is a rare disease with relatively poor outcomes [1, 2]. This cancer accounts for less than 0.1% of all malignancies, and the 5-year overall survival (OS) rate is only 27.1% [2, 3]. Because most previous studies on tracheal cancer were based on small samples [4–7], large sample studies are needed to improve our understanding of this complex disease. In addition, the concepts of health and disease have evolved greatly over the past decades, with a greater emphasis now placed on the role of social determinants in disease development [8, 9]. However, former prognosis analysis mainly focused on pathological and clinical characteristics [10], with few studies addressing the impact of social determinants.

Marital status is an independent prognostic factor for survival in numerous cancers, and married patients have demonstrated better survival in lung cancer, prostate cancer, and breast cancer [11–13]. Marital status is an important type of social support and can greatly affect the patient's mood, quality of life, financial status, and coping strategies [14, 15]. However, our knowledge about its effect on prognosis in tracheal cancer is limited.

In this study, we used data from the Surveillance, Epidemiology, and End Results (SEER) program, which consists of 18 population-based cancer registries covering approximately 30% of the population in the United States. This database provides both clinical records and data on social determinants, including marital status and county level socio-economic data. In this study, we used SEER data to examine the effect of marital status and other prognostic factors on tracheal cancer.

## RESULTS

### Demographic and clinical characteristics

A total of 600 patients in the SEER database from 1990 to 2010 were included in this study. Demographic, clinicopathologic, and treatment data are shown in Table 1. Among the included patients, 344 were married (57.33%), and 256 were unmarried (42.67%), with mean ages of 63.65 ± 12.98 and 63.60 ± 12.98 years, respectively (*p* = 0.963). Compared with unmarried patients, married patients were more likely to be male (62.79% vs. 45.31%, *p* < 0.001). Despite that the race ratio was different between the married and the unmarried group (*p* = 0.005), the proportion of white patients was highest in both groups (79.65% vs. 78.13%). The two groups did not differ significantly with regards to other baseline characteristics, including histologic type, disease extension, tumor grade, selection of surgery, and selection of radiotherapy (*p* > 0.05).

**Table 1:** Baseline demographic and clinical characteristics of patients

	Total	Married	Unmarried^b^	*P* value^c^
	600 (100)	344 (57.33)	256 (42.67)	
**Sex**				< 0.001
Male	332 (55.33)	216 (62.79)	116 (45.31)	
Female	268 (44.67)	128 (37.21)	140 (54.69)	
**Age at diagnosis, y ± SD**	63.63 ± 14.53	63.65 ± 12.98	63.60 ± 12.98	0.963
Race				0.005
White	474 (79.00)	274 (79.65)	200 (78.13)	
Black	77 (12.83)	34 (9.88)	43 (16.80)	
Other	49 (8.17)	36 (10.47)	14 (5.08)	
**Disease Extension**				0.211
Localized	220 (36.67)	134 (38.95)	86 (33.59)	
Regional	205 (34.17)	110 (31.98)	95 (37.11)	
Distant	96 (16.00)	50 (14.53)	46 (17.97)	
Unknown	79 (13.17)	50 (14.53)	29 (11.33)	
**Grade**				0.368
Grade I (well differentiated)	29 (4.83)	14 (4.07)	15 (5.86)	
Grade II (moderately differentiated)	144 (24.00)	78 (22.67)	66 (25.78)	
Grade III(poorly differentiated)	141 (23.50)	82 (23.84)	59 (23.05)	
Grade IV(undifferentiated)	26 (4.33)	19 (5.52)	7 (2.73)	
Unknown	260 (43.33)	151 (43.90)	109 (42.58)	
**Histology**				0.609
Squamous cell carcinoma	299 (49.83)	171 (49.71)	128 (50.00)	
Adenoid cystic carcinoma	107 (17.83)	64 (18.60)	43 (16.80)	
Small cell carcinoma	51 (8.50)	25 (7.27)	26 (10.16)	
Others	143 (23.83)	84 (24.42)	59 (23.05)	
**Low-Grade Malignant Tumors^d^**				0.729
Yes	133 (22.17)	78 (22.67)	55 (21.48)	
No	467 (77.83)	266 (77.33)	201 (78.52)	
**Surgery**				0.744
Total resection	39 (6.50)	25 (7.27)	14 (5.47)	
Subtotal resection	230 (38.33)	134 (38.95)	96 (37.50)	
No cancer directed surgery	323 (53.83)	180 (52.33)	143 (55.86)	
Unknown	8 (1.33)	5 (1.45)	3 (1.17)	
**Radiotherapy**				0.657
Radiotherapy	378 (63.00)	222 (64.53)	156 (60.94)	
No radiotherapy	199 (33.17)	109 (31.69)	90 (35.16)	
Unknown	23 (3.83)	13 (3.78)	10 (3.91)	
**High School Education Rate^e^**				0.806
Lower 50%	327 (54.50)	186 (54.07)	141 (55.08)	
Upper 50%	273 (45.50)	158 (45.93)	115 (44.92)	
**Median Household Income^e^**				0.339
Lower 50%	134 (22.33)	72 (20.93)	62 (24.22)	
Upper 50%	466 (77.77)	272 (79.07)	194 (75.78)	
**Unemployed^e^**				0.890
Lower 50%	309 (51.50)	178 (51.74)	131 (51.17)	
Upper 50%	291 (48.50)	166 (48.26)	125 (48.83)	
**White Collar^e^**				0.787
Lower 50%	184 (30.67)	107 (31.10)	77 (30.08)	
Upper 50%	416 (69.27)	237 (68.90)	179 (69.92)	

a Data were presented as number (percentage) of patients.

b Unmarried group included divorced cases, single (never married) cases, widowed cases and cases separated with spouse.

c *p* values were from the statistical analysis between married and unmarried patients.

d Low-grade malignant tumors were defined as carcinoids, adenoid cystic carcinomas and mucoepidermoid carcinomas.

e Data generated from 2000 national census data. Lower 50% indicated the patients live in counties below median level of national statistics. Upper 50% indicated the patients live in counties above median level of national statistics.

SEER 1990–2010 (*n* = 600)^a^.

### Marital status and overall survival (OS)

**Figure 1:**
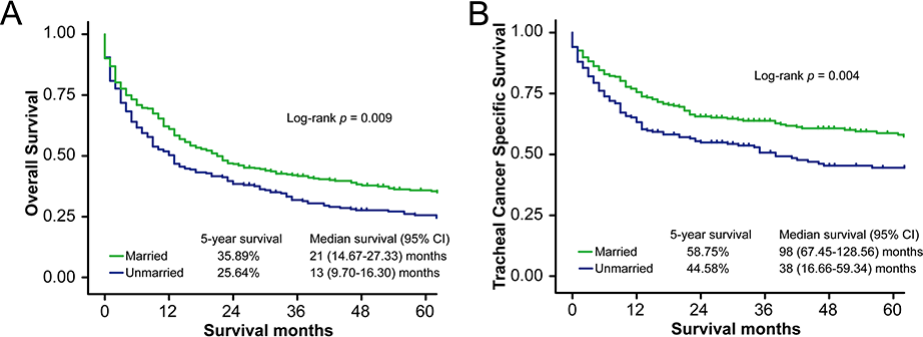
Survival curves in patients with tracheal cancer according to marital status, married vs. unmarried (**A**) Overall survival (OS): χ^2^ = 6.92, *p* = 0.009. (**B**) Tracheal cancer specific survival (TCSS): χ^2^ = 8.11, *p* = 0.004.

The OS curve generated using the Kaplan–Meier method shows a survival difference according to marital status (log-rank test *p* = 0.009) (Figure 1A). The median OS of married patients (21 months, 95% confidence interval [CI] 14.67–27.33 months) was longer than that of unmarried patients (13 months, 95% CI 9.70–16.30 months). Similarly, the 5-year OS rate for married patients (35.89%) was higher than that of unmarried patients (25.64%) (Table 2). Results of the log-rank test showed that age, race, histologic type, tumor grade, disease extension, and treatment (i.e., surgery or radiotherapy) were significantly associated with OS in these patients. After adjusting for these variables and other socioeconomic factors, marital status was found to be an independent prognostic factor, and married patients had better OS compared to unmarried patients (hazard ratio [HR] = 0.78, 95% CI 0.64–0.95, *p* = 0.015). No significant association between county-level socioeconomic status and OS was identified by univariate or multivariate analysis (Table 2).

**Table 2:** Univariate and multivariate analysis for overall survival (OS) in tracheal cancer patients

Variables	5-year OS	Univariate analysis		Multivariate analysis		
		Log rank χ^2^	*P* value	HR	95% CI	*P* value
**Sex**		2.82	0.093			
Female	33.73%			Reference		
Male	29.77%			0.82	0.67–1.01	0.061
**Age at diagnosis**		107.73	< 0.001			
< 65	49.21%			Reference		
≥ 65	14.66%			2.00	1.62–2.46	< 0.001
**Race**		8.88	0.012			
White	28.18%			Reference		
Black	37.96%			0.90	0.67–1.20	0.467
Other	53.92%			0.90	0.60–1.34	0.600
**Histology**		133.81	< 0.001			
Squamous cell carcinoma	18.45%			Reference		
Adenoid cystic carcinoma	75.12%			0.82	0.38–1.75	0.601
Small cell carcinoma	0.00%			1.22	0.86–1.73	0.264
Others	36.98%			0.66	0.50–0.86	0.002
**Low-Grade Malignant Tumors**		109.38	< 0.001			
Yes	72.88%			Reference		
No	19.74%			2.27	1.15–4.47	0.018
**Grade**		23.16	< 0.001			
Grade I (well differentiated)	57.39%			Reference		
Grade II (moderately differentiated)	33.26%			1.27	0.71–2.27	0.416
Grade III (poorly differentiated)	16.74%			1.73	0.97–3.11	0.065
Grade IV (undifferentiated)	17.31%			1.59	0.77–3.26	0.209
Unknown	37.64%			1.35	0.77–2.36	0.294
**Disease Extension**		99.00	< 0.001			
Localized	45.49%			Reference		
Regional	31.24%			1.65	1.29–2.10	< 0.001
Distant	11.01%			2.75	2.06–3.69	< 0.001
Unknown	18.29%			1.50	1.10–2.04	0.009
**Surgery**		167.92	< 0.001			
Surgery-total resection	61.09%			Reference		
Surgery-subtotal resection	52.26%			1.80	1.06–3.05	0.029
No cancer directed surgery	13.33%			3.75	2.20–6.39	< 0.001
Unknown	25.00%			2.72	1.04–7.11	0.041
**Radiotherapy**		7.58	0.023			
Radiotherapy	33.39%			Reference		
No radiotherapy	28.38%			2.06	1.62–2.61	< 0.001
Unknown	27.05%			1.69	0.95–3.03	0.076
**Marital Status**		6.92	0.009			
Unmarried	25.64%			Reference		
Married	35.89%			0.78	0.64–0.95	0.015
**High School Education Rate**		0.74	0.389			
Lower 50%	32.01%			Reference		
Upper 50%	30.89%			0.87	0.65–1.16	0.332
**Median Household Income**		0.66	0.417			
Lower 50%	30.38%			Reference		
Upper 50%	31.94%			1.21	0.92–1.58	0.166
**Unemployed**		1.19	0.275			
Lower 50%	30.80%			Reference		
Upper 50%	32.24%			0.90	0.70–1.17	0.445
**White Collar**		3.57	0.059			
Lower 50%	26.40%			Reference		
Upper 50%	33.77%			0.85	0.66–1.10	0.206

SEER 1990–2010 (*n* = 600).

### Marital status and tracheal cancer specific survival (TCSS)

Median TCSS was also longer for married patients (98 months, 95% CI 67.45–128.56 months) than for unmarried patients (38 months, 95% CI 16.66–59.34 months, log-rank test *p* = 0.004) (Figure 1B). The 5-year TCSS rates for married patients and unmarried patients were 58.75% and 44.58%, respectively (Table 3). Univariate analysis showed that age, race, histologic type, tumor grade, disease extension, and surgery type were significantly associated with TCSS. In addition, patients living in counties with higher median household incomes had better TCSS (5-year TCSS rate 55.81% vs. 42.86%, log-rank test *p* = 0.023). After adjusting for demographic, clinical, and socioeconomic factors, results of multivariate Cox regression analysis confirmed that marital status is an independent prognostic factor, with better TCSS among married patients (HR = 0.70, 95% CI 0.54–0.91, *p* = 0.008). However, multivariate analysis did not support the relationship between county-level household socioeconomic status and TCSS (HR = 0.97, 95% CI 0.69–1.36, *p* = 0.858).

**Table 3:** Univariate and multivariate analysis for tracheal cancer specific survival (TCSS) in tracheal cancer patients

Variables	5-year TCSS	Univariate analysis		Multivariate analysis		
		Log rank χ^2^	*P* value	HR	95% CI	*P* value
**Sex**		2.45	0.118			
Female	55.69%			Reference		
Male	50.38%			0.83	0.63–1.08	0.161
**Age at diagnosis**		23.16	< 0.001			
< 65	61.91%			Reference		
≥ 65	41.27%			1.44	1.10–1.89	0.008
**Race**		7.62	0.022			
White	48.88%			Reference		
Black	66.47%			0.74	0.49–1.13	0.168
Other	67.87%			1.11	0.66–1.86	0.696
**Histology**		88.86	< 0.001			
Squamous cell carcinoma	40.85%			Reference		
Adenoid cystic carcinoma	84.44%			0.84	0.30–2.39	0.750
Small cell carcinoma	0.00%			1.42	0.92–2.19	0.109
Others	60.25%			0.67	0.46–0.97	0.034
**Low-Grade Malignant Tumors**		53.23	< 0.001			
Yes	83.17%			Reference		
No	41.53%			2.25	0.87–5.84	0.094
**Grade**		13.77	0.008			
Grade I (well differentiated)	78.24%			Reference		
Grade II (moderately differentiated)	58.88%			1.12	0.52–2.43	0.773
Grade III (poorly differentiated)	35.44%			1.50	0.69–3.23	0.306
Grade IV (undifferentiated)	32.34%			1.75	0.70–4.41	0.234
Unknown	56.89%			1.47	0.70–3.08	0.305
**Disease Extension**		116.12	< 0.001			
Localized	74.72%			Reference		
Regional	44.97%			2.76	1.94–3.93	< 0.001
Distant	21.50%			4.80	3.23–7.14	< 0.001
Unknown	46.50%			2.20	1.41–3.43	0.001
**Surgery**		94.28	< 0.001			
Surgery-total resection	73.68%			Reference		
Surgery-subtotal resection	71.17%			1.69	0.88–3.27	0.117
No cancer directed surgery	33.50%			3.27	1.69–6.34	< 0.001
Unknown	37.50%			3.49	1.15–10.59	0.028
**Radiotherapy**		1.45	0.484			
Radiotherapy	53.36%			Reference		
No radiotherapy	52.24%			1.80	1.31–2.47	< 0.001
Unknown	56.79%			1.08	0.47–2.49	0.854
**Marital Status**		8.11	0.004			
Unmarried	44.58%			Reference		
Married	58.75%			0.70	0.54–0.91	0.008
**High School Education Rate**		0.96	0.327			
Lower 50%	54.80%			Reference		
Upper 50%	50.43%			1.08	0.74–1.59	0.679
**Median Household Income**		5.10	0.023			
Lower 50%	42.86%			Reference		
Upper 50%	55.81%			0.97	0.69–1.36	0.858
**Unemployed**		0.03	0.863			
Lower 50%	53.26%			Reference		
Upper 50%	52.19%			1.25	0.88–1.76	0.210
**White Collar**		2.50	0.114			
Lower 50%	46.20%			Reference		
Upper 50%	55.62%			1.02	0.72–1.43	0.933

SEER 1990–2010 (*n* = 600).

### Subgroup analysis according to histologic type and tumor stage

**Figure 2:**
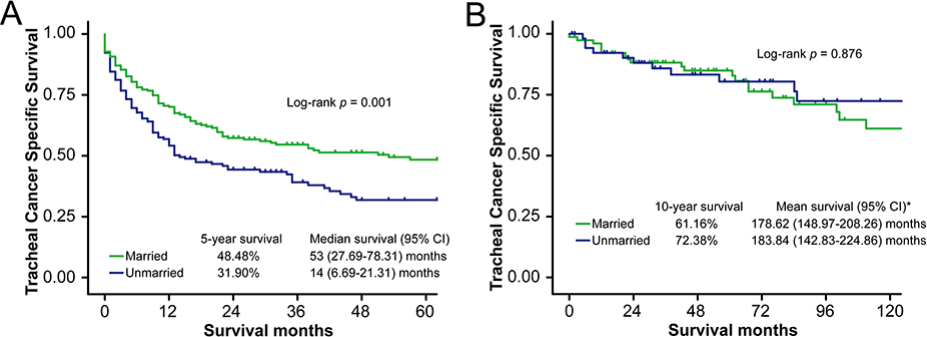
Survival curves in NLGMT and LGMT subgroup patients, married vs. unmarried (**A**) NLGMT subgroup, tracheal cancer specific survival (TCSS): χ^2^ = 10.60, *p* = 0.001. (**B**) LGMT subgroup, tracheal cancer specific survival (TCSS): χ^2^ = 0.02, *p* = 0.876. * No “median survival” calculated in LGMT subgroup because event happened in less than 50% patients at the end of follow-up.

Prognosis of tracheal cancer varies according to histologic type, with low-grade malignant tumors (LGMTs) associated with better outcomes [16]. Therefore, we divided the patients into two subgroups according to histologic type: LGMT vs. non-low-grade malignant tumor (NLGMT). In the NLGMT group, the 5-year TCSS survival rate for married patients (48.48%) was higher than that of unmarried patients (31.90%, log-rank test *p* = 0.001) (Figure 2A, Supplementary Table S1). Results of multivariate analysis showed that marriage was an independent prognostic factor for TCSS in this subgroup (reference: unmarried patients, HR = 0.70, 95% CI 0.53–0.92, *p* = 0.012). However, in the LGMT group, marital status was not associated with TCSS (log-rank test *p* = 0.876, multivariate analysis 0.197) (Figure 2B, Supplementary Table S2). In contrast, no association was found between marital status and OS in either subgroup (Supplementary Tables S3 and S4). Stage-by-stage univariate and multivariate analysis showed that married status was an independent prognostic factor for TCSS in localized disease (reference: unmarried patients, HR = 0.48, 95% CI 0.25–0.91, *p* = 0.023) but not in regional disease or distant metastasis (Supplementary Tables S5 and S6).

## DISCUSSION

In 1977, Engel challenged the traditional “biomedical model” of disease and introduced the “biopsychosocial medical model” [8], which still influences research, medical education, and clinical practice in the 21st century [9]. This model emphasizes viewing the patient as a “whole person” with emotions, feelings, and social context rather than just a disease [17–19].

The importance of marital status and other socioeconomic factors in cancer prognosis have since been recognized. For example, Zhang *et al*. [20] studied 16,910 gastric cancer patients and found that marital status was an independent prognostic factor for both OS and cancer-specific survival. In a study of 129,207 patients, LaPar *et al*. [21] found that socioeconomic factors such as income influenced prognosis after lung cancer surgery. However, because of the rarity of primary tracheal cancer, prognostic factors, especially socioeconomic status, are unclear. Our study found that marital status is an independent prognostic factor for survival in primary tracheal cancer, with better OS and TCSS for married patients than unmarried patients. However, because this was a retrospective study, we could not establish a causal relationship between marital status and survival. Future prospective cohort studies are needed to determine any effect of marriage on survival in patients with tracheal cancer.

Results of subgroup analysis showed that marital status was an independent prognostic factor for TCSS in patients with NLGMTs but not in those with LGMTs. It is possible that patients with LGMTs had less need for social support, given the better prognosis associated with these tumors [16, 22, 23]. Although some studies have found that marriage was more likely to be a protective factor in patients with advanced disease [20, 24, 25], our study failed to prove that marital status played a more important role in late stage patients than in early stage patients. Considering the relatively small size of each subgroup, future studies with larger sample size may be necessary to better test the relationship between marital status and survival according to tumor stage and histologic type.

Numerous studies have investigated the effect of marital status on cancer prognosis. One study evaluating survival among patients with cervical cancer reported an interaction between marital status with tumor stage and treatment [26]. A study on oral and laryngeal cancers suggested that married patients had earlier stage cancer at clinical presentation [27], possibly because a concerned spouse may encourage patients to seek medical help when symptoms are noticed. However, other studies showed no significant relationship between marital status and tumor stage at presentation [20, 28]. In our study, we found no significant difference in disease extension or treatment selection between married and unmarried patients (Table 1). Thus, other explanations are needed to understand the relationship between marital status and prognosis in tracheal cancer.

Emotional state could be one reason. In cancer patients, clinical-level psychological distress was found to be more common in unmarried patients than in married patients, and psychological distress was negatively correlated with social support among married patients [29]. Cancer patients often experience high levels of anxiety and depression [14, 30], and this can affect the immune system through glucocorticoid resistance and decreased interleukin 6 level [31]. In addition, long-term stress can alter the type 1/type 2 cytokine balance, leading to low-grade chronic inflammation and immune cell dysfunction [32], which are related to tumor metastasis [33–35]. In addition, married status generally reflects a better financial situation, which has been reported to improve prognosis [36]. Furthermore, unmarried patients may have additional health care expenditures, as spouses are the major source of outpatient/informal care [37]. Previous studies have found that married patients are more likely to receive better quality clinical care, choose better medical centers, be more compliant with medical recommendations, receive more aggressive treatments, and recover better from surgery, all of which contribute to a better prognosis [38–40].

The effects of other socioeconomic factors have also been discussed in numerous studies. Although most studies suggested that personal income is related to cancer survival [41, 42], the impact of the regional economic environment (e.g., county-level median household income) remains controversial. Higher neighborhood income level has been associated with higher survival rates, which can be partially explained by differences in disease stage at presentation, first-line treatment, and comorbidities [41, 43, 44]. However, other studies reported no significant correlation between county-level income and survival [45] or an association only for patients with early stage disease [46]. Higher education level at the community level has also been identified as having a positive influence on survival [42, 47]; however, our analysis did not show that county-level economic status or education was significant independent prognostic factors. Finally, several studies found that race influenced prognosis for certain types of cancer (e.g., lung, liver, breast, and prostate) [48], whereas other studies found that the effect was minimal after adjusting for other socioeconomic factors [49], which is consistent with our results.

SEER provided us the opportunity to have a relatively large sample size for this rare disease. However, our study still has certain limitations. First, presently SEER does not differentiate between cohabitation (quasi-marriage) and single status; thus, patients who live with their partners may be categorized as unmarried in the SEER database. According to the United States census data, unmarried partners comprise 5.19% of all households [50]. These patients may have better outcomes than unmarried patients who do not live with their partners, potentially biasing our results towards the null. This factor should be taken into consideration, and more data regarding cohabitation and quasi-marriage status is needed in future studies. Second, the database did not provide information related to sexual minority status (lesbian, gay, bisexual, or transgender). Several studies have suggested that these patients may have different types of social support and prognostic factors [51–53]. Thus, future studies on the relationship between marital status and cancer prognosis should take sexual orientation into account. Third, the database did not provide details about the quality of the marriage (e.g., satisfaction with the relationship, sexual activity), which limits the ability to analyze the impact of marriage on prognosis in tracheal cancer. In addition, the study did not analyze changes in marital status, length of the marriage, duration of being single, lifestyle risk factors, or individual-level socioeconomic factors. As the final comment, several important clinical or treatment factors that may affect survival is also not registered in SEER, including details of surgery or treatment of chemotherapy and quality of treatment at various institutions. To verify our results, further studies with detailed data at both the county and individual levels are warranted.

In conclusion, our study based on the SEER database shows that marital status is an independent prognostic factor for survival in patients with tracheal cancer. To improve prognosis, additional social support should be considered for unmarried patients, especially those with NLGMTs. The impact of other individual-level socioeconomic factors on survival requires further study.

## MATERIALS AND METHODS

### Data sources

All primary data were obtained by using SEER*Stat software version 8.3.2 and the SEER database released in April 2015. County-level socioeconomic information for the year 2000 was obtained from US Census 2000 files, made available by the US Census Bureau and linked to the SEER database.

### Inclusion and exclusion criteria

Information about patients with malignancies of the “trachea, mediastinum, and other respiratory organs” from January 1990 to December 2010 was extracted from the SEER database. Patients with histologically confirmed primary tracheal cancer (International Classification of Diseases for Oncology, Third Edition [ICD-O-3], code C33.9) were considered for analysis. Exclusion criteria were incomplete follow-up information, unknown survival time or survival time of 0 days, age < 18 years, unknown race, and unknown marital status (Supplementary Figure S1).

### Study variables

Clinically related information extracted from the SEER database included sex, age, race, primary tumor site, histologic type, tumor grade, disease extension, surgical record, and radiotherapy. Patients were divided into two groups according to age (< 65 years vs. ≥ 65 years). Race was classified as white, black, or other. Histologic type was further classified as squamous cell carcinoma, adenoid cystic carcinoma, small cell carcinoma, or other. Histologic type was further classified as LGMT vs. NLGMT, as previously described [16], with carcinoids, adenoid cystic carcinomas, and mucoepidermoid carcinomas defined as LGMTs, and all other histologic types defined as NLGMTs. Disease extension was categorized using the Collaborative Stage classification criteria, with “localized” indicating situ lesions or lesions confined to the organ of origin, “regional” indicating invasion into surrounding organs/adjacent tissues or regional lymph node metastasis, and “distant” indicating distant metastasis. The surgical record was categorized as complete, partial, none, or unknown, and procedures were listed as “radical surgery” or “total surgical removal of primary site”; “local tumor destruction” or “local tumor excision”; “simple/partial surgical removal of primary site” or “debulking”; “none”; or “unknown” in the SEER database.

Marital status was categorized as married or unmarried (including never married single, divorced, separated, and widowed). Socioeconomic factors included the following county attributes: high school education rate, median household income, unemployment rate, and proportion of white-collar workers in the population. All county attributes variables were divided into two groups by the median number.

### Outcomes

The primary outcomes were OS and TCSS. OS was defined as the time from diagnosis to date of death from any cause. Patients who were alive on the date of last contact or at the follow-up cut-off date were censored. TCSS was defined as the time from diagnosis to date of death due to tracheal cancer. Death caused by tracheal cancer was considered an event. Patients who died from other causes or were still alive at the end of the study were censored. The follow-up cut-off date was December 31, 2012, according to the SEER database instructions.

### Statistical analyses

Demographic, clinical, and socioeconomic characteristics were reported as means and standard deviations for continuous variables and percentages for categorical variables. Differences in baseline characteristics were compared by Pearson chi-squared test for categorical variables and *t*-test for continuous variables. OS and TCSS were calculated by the Kaplan–Meier method and log-rank tests were conducted to compare differences between subgroups of each variable. Multivariate Cox proportional hazard models were used to determine risk factors that affect survivorship. All *P* values were two-sided, and a value less than 0.05 were considered significant. All data were analyzed by SPSS version 20 (Statistics Package for Social Science, Chicago, IL).

## SUPPLEMENTARY MATERIALS METHODS FIGURE AND TABLES




