# Supplementary material for: Marital status is an independent prognostic factor for tracheal cancer patients: an analysis of the SEER database
Source: Oncotarget. 2016 Oct 21;7(47):77152–62. doi: 10.18632/oncotarget.12809 (PMC5363576; doi:10.18632/oncotarget.12809)
Supplement: Supplementary file 1 [file oncotarget-07-77152-s001.pdf]

Marital status is an independent prognostic factor for tracheal cancer patients: an analysis of the SEER database

Supplementary Materials

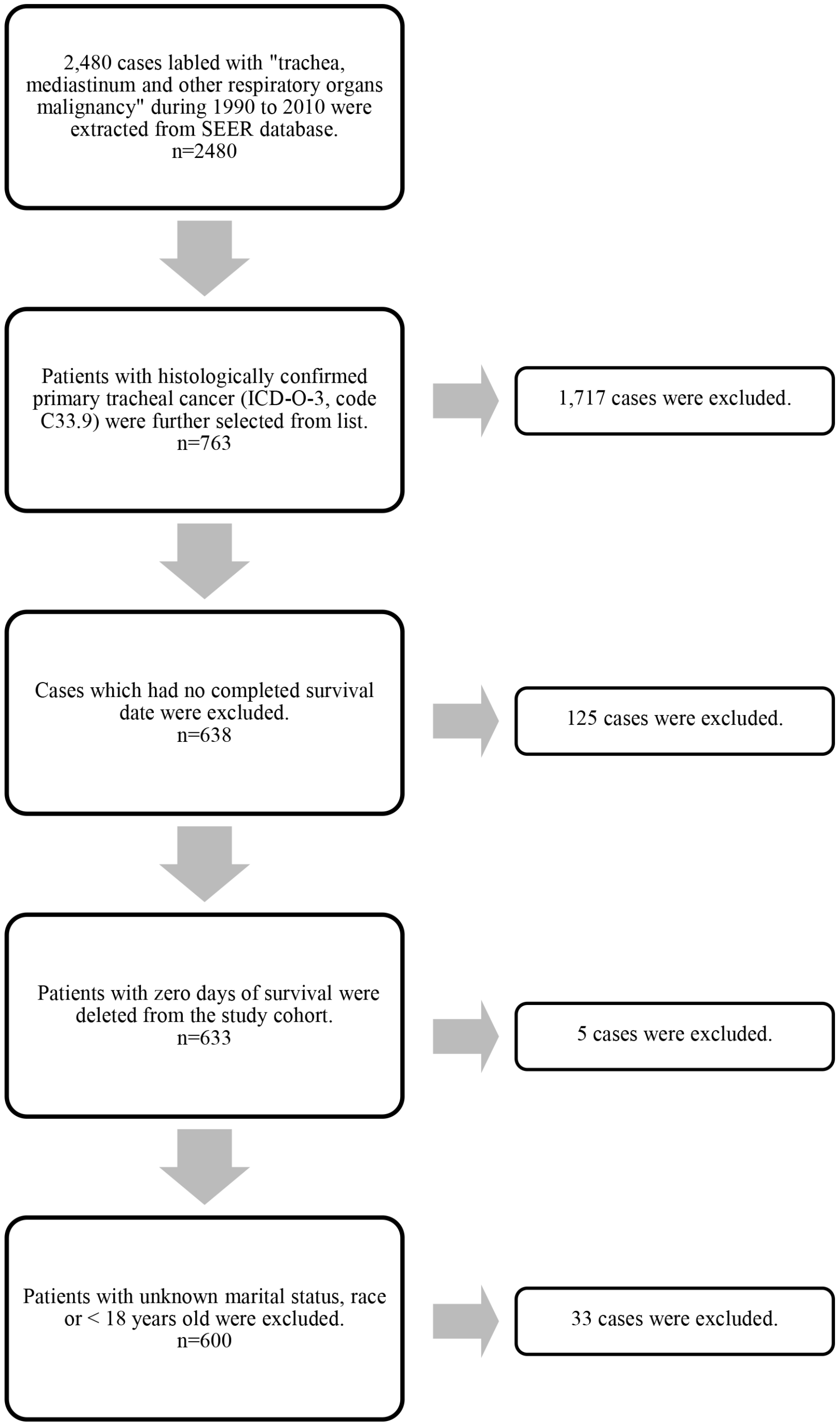

Figure 1: Flow chart of patient selection.

Supplementary Table S1: Univariate and multivariate survival analysis of tracheal cancer specific survival (TCSS) in non-low grade malignant tracheal cancer patients. See Supplementary_Table_S1

Supplementary Table S2: Univariate and multivariate survival analysis of TCSS in low grade malignant tracheal cancer patients. See Supplementary_Table_S2

Supplementary Table S3: Univariate and multivariate survival analysis of overall survival (OS) in low grade malignant tracheal cancer patients. See Supplementary_Table_S3

Supplementary Table S4: Univariate and multivariate survival analysis of OS in non-low grade malignant tracheal cancer patients. See Supplementary_Table_S4

Supplementary Table S5: Univariate and multivariate survival analysis for marital status on OS by stage at diagnosis

SEER stage	5-year OS	Univariate analysis		Multivariate analysis[a]		
		Log rank χ^2	*P* value	HR	95% CI	*P* value
**Localized (*n* = 220)**						
**Marital status**		2.33	0.127			
Unmarried	38.81%			Reference		
Married	50.03%			0.73	0.49–1.10	0.131
**Regional (*n* = 205)**						
**Marital status**		2.85	0.092			
Unmarried	25.91%			Reference		
Married	35.44%			0.78	0.55–1.11	0.164
**Distant (*n* = 96)**						
**Marital status**		1.29	0.256			
Unmarried	9.13%			Reference		
Married	12.34%			0.76	0.47–1.25	0.277

[a]Factors including gender, age, race, histological type, grade, treatment (surgery and radiotherapy), and other socio-economic factors (education, income, employment) had been adjusted in each stage.
SEER 1990-2010 (*n* = 521).

Supplementary Table S6: Univariate and multivariate survival analysis for marital status on TCSS by stage at diagnosis

SEER stage	5-year TCSS	Univariate analysis		Multivariate analysisa		
		Log rank χ^2	*P* value	HR	95% CI	*P* value
**Localized (*n* = 220)**						
**Marital status**		3.38	0.066			
Unmarried	66.51%			Reference		
Married	80.04%			0.48	0.25–0.91	0.023
**Regional(*n* = 205)**						
**Marital status**		2.79	0.095			
Unmarried	37.79%			Reference		
Married	50.67%			0.70	0.46–1.07	0.098
**Distant(*n* = 96)**						
**Marital status**		1.03	0.309			
Unmarried	16.79%			Reference		
Married	25.33%			0.73	0.41	1.31

[a]Factors including gender, age, race, histological type, grade, treatment (surgery and radiotherapy), and other socio-economic factors (education, income, employment) had been adjusted in each stage.
SEER 1990-2010 (*n* = 521).
