# Supplementary material for: Marital status is an independent prognostic factor for tracheal cancer patients: an analysis of the SEER database
Source: Oncotarget. 2016 Oct 21;7(47):77152–62. doi: 10.18632/oncotarget.12809 (PMC5363576; doi:10.18632/oncotarget.12809)
Supplement: Supplementary file 2 [file oncotarget-07-77152-s002.docx]

| **Supplementary Table 1: Univariate and multivariate survival analysis of TCSS in non-low grade malignant tracheal cancer patients. SEER 1990-2010 (n=467)** | | | | | | | | | |
| --- | --- | --- | --- | --- | --- | --- | --- | --- | --- |
| **Variables** | **5-year TCSS** | **Univariate analysis** | | | **Multivariate analysis** | | | | |
|  |  | **Log rank χ^2^** | **P value** | | **HR** | **95% CI** | | **P value** | |
| **Sex** |  | 0.02 | 0.894 | |  |  | |  | |
| Female | 41.78% |  |  | | Reference |  | |  | |
| Male | 41.39% |  |  | | 0.78 | 0.59-1.03 | | 0.078 | |
| **Age at diagnosis** |  | 9.31 | 0.002 | |  |  | |  | |
| <65 | 47.54% |  |  | | Reference |  | |  | |
| ≥65 | 36.10% |  |  | | 1.42 | 1.07-1.90 | | 0.016 | |
| **Race** |  | 4.10 | 0.129 | |  |  | |  | |
| White | 38.43% |  |  | | Reference |  | |  | |
| Black | 59.24% |  |  | | 0.73 | 0.47-1.13 | | 0.156 | |
| Other | 41.21% |  |  | | 1.15 | 0.60-2.20 | | 0.669 | |
| **Histology** |  | 29.85 | <0.001 | |  |  | |  | |
| Squamous cell carcinoma | 40.85% |  |  | | Reference |  | |  | |
| Small cell carcinoma | 0.00% |  |  | | 1.45 | 0.95-2.23 | | 0.087 | |
| Others | 56.25% |  |  | | 0.68 | 0.47-0.98 | | 0.041 | |
| **Grade** |  | 8.36 | 0.079 | |  |  | |  | |
| Grade I (well differentiated) | 60.81% |  |  | | Reference |  | |  | |
| Grade II (moderately differentiated) | 51.67% |  |  | | 0.92 | 0.38-2.23 | | 0.848 | |
| Grade III (poorly differentiated) | 33.37% |  |  | | 1.13 | 0.47-2.70 | | 0.789 | |
| Grade IV (undifferentiated) | 25.89% |  |  | | 1.31 | 0.47-3.62 | | 0.601 | |
| Unknown | 41.91% |  |  | | 1.12 | 0.47-2.68 | | 0.796 | |
| **Disease Extension** |  | 90.55 | <0.001 | |  |  | |  | |
| Localized | 67.11% |  |  | | Reference |  | |  | |
| Regional | 31.39% |  |  | | 2.97 | 1.99-4.44 | | <0.001 | |
| Distant | 15.79% |  |  | | 5.34 | 3.47-8.22 | | <0.001 | |
| Unknown | 39.88% |  |  | | 2.43 | 1.51-3.90 | | <0.001 | |
| **Surgery** |  | 47.92 | <0.001 | |  |  | |  | |
| Yes | 60.83% |  |  | | Reference |  | |  | |
| No Known cancer directed surgery | 28.94% |  |  | | 2.18 | 1.54-3.08 | | <0.001 | |
| **Radiotherapy, RT** |  | 3.65 | 0.056 | |  |  | |  | |
| Yes | 43.08% |  |  | | Reference |  | |  | |
| No known RT | 39.38% |  |  | | 1.78 | 1.28-2.48 | | 0.001 | |
|  |  |  |  | |  |  | |  | |
|  |  |  |  | |  |  | | *(Continued)* | |
| **Variables** | **5-year TCSS** | **Univariate analysis** | | | **Multivariate analysis** | | | | |
|  |  | **Log rank χ^2^** | | **P value** | **HR** | | **95% CI** | | **P value** |
| **Marital Status** |  | 10.60 | | 0.001 |  | |  | |  |
| Unmarried | 31.90% |  | |  | Reference | |  | |  |
| Married | 48.48% |  | |  | 0.70 | | 0.53-0.93 | | 0.012 |
| **High School Education Rate** |  | 0.25 | | 0.618 |  | |  | |  |
| Lower 50% | 44.40% |  | |  | Reference | |  | |  |
| Upper 50% | 38.70% |  | |  | 1.10 | | 0.74-1.65 | | 0.637 |
| **Median Household Income** |  | 2.63 | | 0.105 |  | |  | |  |
| Lower 50% | 35.02% |  | |  | Reference | |  | |  |
| Upper 50% | 43.97% |  | |  | 0.89 | | 0.62-1.27 | | 0.503 |
| **Unemployed** |  | 0.46 | | 0.499 |  | |  | |  |
| Lower 50% | 42.68% |  | |  | Reference | |  | |  |
| Upper 50% | 40.05% |  | |  | 1.30 | | 0.90-1.87 | | 0.161 |
| **White Collar** |  | 0.29 | | 0.589 |  | |  | |  |
| Lower 50% | 36.95% |  | |  | Reference | |  | |  |
| Upper 50% | 43.77% |  | |  | 1.15 | | 0.80-1.65 | | 0.464 |

| **Supplementary Table 2: Univariate and multivariate survival analysis of tracheal cancer specific survivals (TCSS) in low grade malignant tracheal cancer patients. SEER 1990-2010 (n=133)** | | | | | | |
| --- | --- | --- | --- | --- | --- | --- |
| **Variables** | **10-year TCSS** | **Univariate analysis** | | **Multivariate analysis** | | |
|  |  | **Log rank χ^2^** | **P value** | **HR** | **95% CI** | **P value** |
| **Sex** |  | 0.18 | 0.669 |  |  |  |
| Female | 70.70% |  |  | Reference |  |  |
| Male | 55.87% |  |  | 1.03 | 0.42-2.51 | 0.952 |
| **Age at diagnosis** |  | 061 | 0.434 |  |  |  |
| <65 | 65.80% |  |  | Reference |  |  |
| ≥65 | 70.15% |  |  | 1.50 | 0.61-3.72 | 0.376 |
| **Race** |  | 0.67 | 0.717 |  |  |  |
| White | 64.23% |  |  | Reference |  |  |
| Black | 73.84% |  |  | 0.71 | 0.11-4.52 | 0.713 |
| Other | 68.00% |  |  | 1.08 | 0.42-2.75 | 0.877 |
| **Histology** |  | 6.31 | 0.043 |  |  |  |
| Adenoid cystic carcinoma | 63.59% |  |  | Reference |  |  |
| Carcinoids | 50.00% |  |  | 2.04 | 0.43-9.55 | 0.367 |
| Mucoepidermoid carcinoma | 92.86% |  |  | 0.17 | 0.01-2.92 | 0.221 |
| **Grade** |  | 4.20 | 0.380 |  |  |  |
| Grade I (well differentiated) | 55.56% |  |  | Reference |  |  |
| Grade II (moderately differentiated) | 90.00% |  |  | 1.01 | 0.10-9.85 | 0.992 |
| Grade III (poorly differentiated) | 66.67% |  |  | 1.15 | 0.12-11.43 | 0.904 |
| Grade IV (undifferentiated) | 75.00% |  |  | 4.35 | 0.10-194.68 | 0.448 |
| Unknown | 62.88% |  |  | 1.35 | 0.28-6.38 | 0.709 |
| **Disease Extension** |  | 5.25 | 0.154 |  |  |  |
| Localized | 74.21% |  |  | Reference |  |  |
| Regional | 53.54% |  |  | 1.31 | 0.58-2.95 | 0.515 |
| Distant | 41.67% |  |  | 1.23 | 0.21-7.19 | 0.818 |
| Unknown^a^ | 100.00% |  |  | n/a | n/a | 0.977 |
| **Surgery** |  | 5.42 | 0.020 |  |  |  |
| Yes | 70.41% |  |  | Reference |  |  |
| No Known cancer directed surgery | 25.40% |  |  | 2.45 | 0.99-6.06 | 0.053 |
|  |  |  |  |  |  |  |
|  |  |  |  |  |  |  |
|  |  |  |  |  |  | *(Continued)* |
| **Variables** | **10-year TCSS** | **Univariate analysis** | | **Multivariate analysis** | | |
|  |  | **Log rank χ^2^** | **P value** | **HR** | **95% CI** | **P value** |
| **Radiotherapy, RT** |  | 2.99 | 0.084 |  |  |  |
| Yes | 59.95% |  |  | Reference |  |  |
| No known RT | 78.05% |  |  | 0.70 | 0.25-2.01 | 0.511 |
| **Marital Status** |  | 0.02 | 0.876 |  |  |  |
| Unmarried | 72.38% |  |  | Reference |  |  |
| Married | 61.16% |  |  | 0.56 | 0.24-1.35 | 0.197 |
| **High School Education Rate** |  | 0.31 | 0.576 |  |  |  |
| Lower 50% | 67.51% |  |  | Reference |  |  |
| Upper 50% | 63.29% |  |  | 0.78 | 0.23-2.82 | 0.732 |
| **Median Household Income** |  | 1.41 | 0.235 |  |  |  |
| Lower 50% | 87.50% |  |  | Reference |  |  |
| Upper 50% | 63.35% |  |  | 6.82 | 1.09-42.64 | 0.040 |
| **Unemployed** |  | 0.54 | 0.459 |  |  |  |
| Lower 50% | 63.80% |  |  | Reference |  |  |
| Upper 50% | 67.25% |  |  | 0.85 | 0.26-2.81 | 0.791 |
| **White Collar** |  | 2.07 | 0.151 |  |  |  |
| Lower 50% | 54.43% |  |  | Reference |  |  |
| Upper 50% | 69.57% |  |  | 0.30 | 0.10-0.89 | 0.029 |
| ^a^ Only 6 patients in this subgroup who were all alive at the follow-up cut-off date. | | | | | | |

| **Supplementary Table 3: Univariate and multivariate survival analysis of overall survival (OS) in low grade malignant tracheal cancer patients. SEER 1990-2010 (n=133)** | | | | | | | | |
| --- | --- | --- | --- | --- | --- | --- | --- | --- |
| **Variables** | **10-year OS** | **Univariate analysis** | | **Multivariate analysis** | | | | |
|  |  | **Log rank χ^2^** | **P value** | **HR** | **95% CI** | | **P value** | |
| **Sex** |  | 0.20 | 0.652 |  |  | |  | |
| Female | 55.01% |  |  | Reference |  | |  | |
| Male | 52.29% |  |  | 1.11 | 0.56-2.19 | | 0.774 | |
| **Age at diagnosis** |  | 16.14 | <0.001 |  |  | |  | |
| <65 | 61.65% |  |  | Reference |  | |  | |
| ≥65 | 34.55% |  |  | 3.02 | 1.56-5.85 | | 0.001 | |
| **Race** |  | 0.45 | 0.799 |  |  | |  | |
| White | 54.78% |  |  | Reference |  | |  | |
| Black | 60.95% |  |  | 1.56 | 0.43-5.63 | | 0.500 | |
| Other | 52.53% |  |  | 1.19 | 0.56-2.52 | | 0.656 | |
| **Histology** |  | 5.59 | 0.061 |  |  | |  | |
| Adenoid cystic carcinoma | 52.84% |  |  | Reference |  | |  | |
| Carcinoids | 40.91% |  |  | 0.65 | 0.19-2.30 | | 0.508 | |
| Mucoepidermoid carcinoma | 78.79% |  |  | 0.40 | 0.08-2.15 | | 0.288 | |
| **Grade** |  | 18.39 | 0.001 |  |  | |  | |
| Grade I (well differentiated) | 50.93% |  |  | Reference |  | |  | |
| Grade II (moderately differentiated) | 85.71% |  |  | 0.81 | 0.14-4.64 | | 0.816 | |
| Grade III (poorly differentiated) | 33.33% |  |  | 5.10 | 0.93-27.86 | | 0.060 | |
| Grade IV (undifferentiated) | 0.00% |  |  | 3.52 | 0.43-28.98 | | 0.242 | |
| Unknown | 53.99% |  |  | 1.39 | 0.38-5.06 | | 0.615 | |
| **Disease Extension** |  | 5.93 | 0.115 |  |  | |  | |
| Localized | 64.12% |  |  | Reference |  | |  | |
| Regional | 43.58% |  |  | 1.70 | 0.86-3.35 | | 0.124 | |
| Distant | 35.71% |  |  | 2.43 | 0.62-9.42 | | 0.201 | |
| Unknown | 66.67% |  |  | 0.22 | 0.03-1.60 | | 0.135 | |
|  |  |  |  |  |  | |  | |
|  |  |  |  |  |  | |  | |
|  |  |  |  |  |  | |  | |
|  |  |  |  |  |  | |  | |
|  |  |  |  |  |  | | | |
|  |  |  |  |  | *(Continued)* | | | |
| **Variables** | **10-year OS** | **Univariate analysis** | | **Multivariate analysis** | | | | |
|  |  | **Log rank χ^2^** | **P value** | **HR** | | **95% CI** | | **P value** |
| **Surgery** |  | 15.26 | <0.001 |  | |  | |  |
| Yes | 61.37% |  |  | Reference | |  | |  |
| No Known cancer directed surgery | 17.38% |  |  | 3.35 | | 1.61-6.97 | | 0.001 |
| **Radiotherapy, RT** |  | 0.12 | 0.725 |  | |  | |  |
| Yes | 51.84% |  |  | Reference | |  | |  |
| No known RT | 60.00% |  |  | 1.59 | | 0.74-3.41 | | 0.235 |
| **Marital Status** |  | 1.51 | 0.219 |  | |  | |  |
| Unmarried | 53.75% |  |  | Reference | |  | |  |
| Married | 54.36% |  |  | 0.47 | | 0.23-0.92 | | 0.028 |
| **High School Education Rate** |  | 0.08 | 0.775 |  | |  | |  |
| Lower 50% | 55.11% |  |  | Reference | |  | |  |
| Upper 50% | 53.46% |  |  | 0.71 | | 0.28-1.78 | | 0.465 |
| **Median Household Income** |  | 1.13 | 0.287 |  | |  | |  |
| Lower 50% | 65.88% |  |  | Reference | |  | |  |
| Upper 50% | 53.18% |  |  | 2.54 | | 0.78-8.30 | | 0.124 |
| **Unemployed** |  | 0.81 | 0.368 |  | |  | |  |
| Lower 50% | 52.11% |  |  | Reference | |  | |  |
| Upper 50% | 56.41% |  |  | 0.61 | | 0.26-1.41 | | 0.244 |
| **White Collar** |  | 0.70 | 0.401 |  | |  | |  |
| Lower 50% | 42.84% |  |  | Reference | |  | |  |
| Upper 50% | 58.00% |  |  | 0.49 | | 0.21-1.13 | | 0.095 |

| **Supplementary Table 4: Univariate and multivariate survival analysis of OS in non-low grade malignant tracheal cancer patients. SEER 1990-2010 (n=467)** | | | | | | |
| --- | --- | --- | --- | --- | --- | --- |
| **Variables** | **5-year OS** | **Univariate analysis** | | **Multivariate analysis** | | |
|  |  | **Log rank χ^2^** | **P value** | **HR** | **95% CI** | **P value** |
| **Sex** |  | 0.12 | 0.729 |  |  |  |
| Female | 18.57% |  |  | Reference |  |  |
| Male | 20.53% |  |  | 0.81 | 0.65-1.00 | 0.047 |
| **Age at diagnosis** |  | 51.48 | <0.001 |  |  |  |
| <65 | 31.81% |  |  | Reference |  |  |
| ≥65 | 11.08% |  |  | 1.93 | 1.55-2.41 | <0.001 |
| **Race** |  | 2.68 | 0.262 |  |  |  |
| White | 17.69% |  |  | Reference |  |  |
| Black | 29.63% |  |  | 0.90 | 0.66-1.22 | 0.507 |
| Other | 25.51% |  |  | 0.84 | 0.51-1.38 | 0.488 |
| **Histology** |  | 26.19 | <0.001 |  |  |  |
| Squamous cell carcinoma | 18.45% |  |  | Reference |  |  |
| Small cell carcinoma | 0.00% |  |  | 1.31 | 0.93-1.85 | 0.124 |
| Others | 31.09% |  |  | 0.67 | 0.51-0.88 | 0.004 |
| **Grade** |  | 5.76 | 0.218 |  |  |  |
| Grade I (well differentiated) | 32.27% |  |  | Reference |  |  |
| Grade II (moderately differentiated) | 24.05% |  |  | 1.00 | 0.52-1.91 | 0.998 |
| Grade III (poorly differentiated) | 15.71% |  |  | 1.22 | 0.64-2.34 | 0.539 |
| Grade IV (undifferentiated) | 18.18% |  |  | 0.99 | 0.45-2.16 | 0.977 |
| Unknown | 19.18% |  |  | 0.99 | 0.52-1.88 | 0.973 |
| **Disease Extension** |  | 61.90 | <0.001 |  |  |  |
| Localized | 30.67% |  |  | Reference |  |  |
| Regional | 18.78% |  |  | 1.51 | 1.15-1.97 | 0.003 |
| Distant | 6.95% |  |  | 2.69 | 1.99-3.63 | <0.001 |
| Unknown | 14.50% |  |  | 1.44 | 1.05-1.97 | 0.024 |
|  |  |  |  |  |  |  |
|  |  |  |  |  |  |  |
|  |  |  |  |  |  |  |
|  |  |  |  |  |  |  |
|  |  |  |  |  |  |  |
|  |  |  |  |  |  |  |
|  |  |  |  |  |  | *(Continued)* |
| **Variables** | **5-year OS** | **Univariate analysis** | | **Multivariate analysis** | | |
|  |  | **Log rank χ^2^** | **P value** | **HR** | **95% CI** | **P value** |
| **Surgery** |  | 77.17 | <0.001 |  |  |  |
| Yes | 37.18% |  |  | Reference |  |  |
| No Known cancer directed surgery | 10.49% |  |  | 2.23 | 1.73-2.87 | <0.001 |
| **Radiotherapy, RT** |  | 11.39 | 0.001 |  |  |  |
| Yes | 21.79% |  |  | Reference |  |  |
| No known RT | 16.27% |  |  | 1.99 | 1.56-2.54 | <0.001 |
| **Marital Status** |  | 6.47 | 0.011 |  |  |  |
| Unmarried | 15.02% |  |  | Reference |  |  |
| Married | 23.20% |  |  | 0.83 | 0.67-1.02 | 0.080 |
| **High School Education Rate** |  | 0.08 | 0.773 |  |  |  |
| Lower 50% | 19.97% |  |  | Reference |  |  |
| Upper 50% | 19.47% |  |  | 0.86 | 0.63-1.16 | 0.326 |
| **Median Household Income** |  | 0.10 | 0.752 |  |  |  |
| Lower 50% | 22.79% |  |  | Reference |  |  |
| Upper 50% | 18.84% |  |  | 1.18 | 0.90-1.56 | 0.233 |
| **Unemployed** |  | 0.30 | 0.583 |  |  |  |
| Lower 50% | 19.95% |  |  | Reference |  |  |
| Upper 50% | 19.46% |  |  | 0.90 | 0.68-1.19 | 0.461 |
| **White Collar** |  | 0.62 | 0.433 |  |  |  |
| Lower 50% | 17.21% |  |  | Reference |  |  |
| Upper 50% | 21.00% |  |  | 0.88 | 0.67-1.15 | 0.334 |
